# Victimization from bullying among school-attending adolescents in grades 7 to 10 in Zambia

**DOI:** 10.5249/jivr.v4i1.84

**Published:** 2012-01

**Authors:** Seter Siziya, Emmanuel Rudatsikira, Adamson S. Muula

**Affiliations:** ^*a*^Department of Community Medicine, School of Medicine, University of Zambia, Lusaka, Zambia.; ^*b*^School of Community and Environmental Health, Old Dominion University, Norfolk, Virginia, United States.; ^*c*^Department of Public Health, College of Medicine, University of Malawi, Blantyre, Malawi.

## Abstract

**Background:**

Among school- attending adolescents, victimization from bullying is associated with anxiety, depression and poor academic performance. There are limited reports on victimization from bullying in Zambia; we therefore conducted this study to determine the prevalence and correlates for victimization from bullying among adolescents in grades 7 to 10 in the country in order to add information on the body of knowledge on victimization from bullying.

**Methods:**

The 2004 Zambia Global School-based Health Survey (GSHS) data among adolescents in grades 7 to 10 were obtained from the World Health Organization. We estimated the prevalence of victimization from bullying. We also conducted weighted multivariate logistic regression analysis to determine independent factors associated with victimization from bullying, and report adjusted odds ratios (AOR) and their 95% confidence intervals (CI).

**Results:**

Of 2136 students who participated in the 2004 Zambia GSHS, 1559 had information on whether they were bullied or not. Of these, 1559 students, 62.8% (60.0% of male and 65.0% of female) participants reported having been bullied in the previous 30 days to the survey. We found that respondents of age less than 14 years were 7% (AOR=0.93; 95%CI [0.91, 0.95]) less likely to have been bullied compared to those aged 16 years or older. Being a male (AOR=1.07; 95%CI [1.06, 1.09]), lonely (AOR=1.24; 95%CI [1.22, 1.26]), worried (AOR=1.12; 95%CI [1.11, 1.14]), consuming alcohol (AOR=2.59; 95%CI [2.55, 2.64]), missing classes (AOR=1.30; 95%CI [1.28, 1.32]), and considering attempting suicide (AOR=1.20; 95%CI [1.18, 1.22]) were significantly associated with bullying victimization.

**Conclusions:**

Victimization from bullying is prevalent among in-school adolescents in grades 7 to 10 in Zambia, and interventions to curtail it should consider the factors that have been identified in this study.

## Introduction

Victimization from bullying among school-aged children is a public health problem. It is associated with emotional, behavioral and social problems such as tobacco, alcohol, or drug use, sexual intercourse,^1^depression,^2-6^ physical fighting,^7^self harm,^8^attempted and completed suicide,^9,10^suicide ideation,^1^and truancy.^11^

Although bullying is a universal phenomenon, there are significant regional variations in rates of victimization. For example, in a study conducted in 40 countries across European, North American countries and Israel, Craig et al found that Baltic countries had higher rates of victimization while northern European countries had the lowest rates.^12^

In another study on the prevalence of victimization from bullying among 13-15 year-old school children in 66 countries from five continents, Due et al^13^reported that the lowest prevalence was from Tajikistan (7.1% for boys and girls), and the highest rate for boys was found in Zimbabwe, Bulawayo (70.2%), and for girls in Zambia (67.1%).

Results on the correlates for victimization from bullying have not been consistent, especially for the factors: sex and age. Craig et al ^12^reported that the rate of victimization decreased with an increase in age for boys in 30 of 40 countries, and for girls in 25 of 39 countries. The rest of the associations were either showing an increase in the rate of victimization with respect to age or no significant trend was observed in the victimization rate over age.

Studies conducted using the 2004 Zambia Global School-based Health Survey (GSHS) data by Due et al^13^and Fleming and Jacobsen^1^were restricted to pupils of ages 13-15 years: pupils younger than 13 years (10.7%) and older than 15 years (30.4%) in the sampled school Grades were excluded from the study. Therefore, the reported prevalence for victimization from bullying may have been a biased estimate for the true prevalence among students in grades 7 to 10.

Although plenty of literature exists on the associations of loneliness, having worried thoughts, alcohol consumption, and attempting suicide on one hand and victimization from bullying on the other, the patterns of these behaviors may be different between countries or/and cultures.^14^However, Fleming and Jacobsen,^1^using the 2004 Zambia GSHS data reported some demographic, individual and behavioral correlates for victimization from bullying among the 13-15-year olds but did not report on the association between truancy and being bullied. Moreover, this study reduced the sample size of adolescents in grades 7-10 by excluding adolescents who were of age less than 13 years and those older than 15 years, thus, reducing the statistical power of the study, especially for the factor: age. Furthermore, exclusion of some adolescents from grades 7-10 may have affected the generalizability of the findings from this study to the target school going adolescents’ population in grades 7-10.

It is important that country specific correlates for victimization be determined in view of the inconsistent findings reported above. In view of the above discussion, we conducted this study using data from the 2004 Zambia GSHS in order to estimate the prevalence of victimization amongst all pupils in the sampled grades 7 to 10, irrespective of their ages; and to determine correlates for victimization with increased statistical power that may be generalized to the school-attending adolescent population at a national level in Zambia. Data on the prevalence and associated factors for victimization may aid in the design of interventions aimed at preventing and controlling this problem among school-attending adolescents.

## Methods

Details of the methodology for the 2004 Zambia GSHS are described elsewhere.^15^However, in brief, the GSHS was a school-based cross-sectional national study that was conducted among students in grades 7 to 10 with the aim of capturing most of the adolescents in the 13-15 years age group. 

**Sampling**

The 2004 Zambia GSHS used a two-stage probability sampling technique. In the first stage of sampling, the primary sampling units were schools that were selected with a probability proportional to their enrolment size. In the second step, a systematic sample of classes in the selected school was obtained. All students in the selected classes in grades 7-10 were eligible to participate. Out of 3021 adolescents who were in grades 7–10, 2257 participated in the survey, giving a response rate of 75%. Students who were not available at their school on the day the survey was conducted at their school were not followed-up. No reasons were sought in the case of those absent. Thus, the sample available for our study comprised all adolescents in grades 7 to 10 who were present during the survey, irrespective of their ages.

**Ethical considerations**

Permission to carry out the survey was obtained from the Ministries of Health and Education. Informed consent to participate in the study was collected from students aged 16 years or older; and parental consent was obtained for those younger than 16 years. The school managers gave permission to conduct the survey. Confidentiality was upheld by allowing for anonymity in completing the questionnaire.

**Questionnaire completion**

The questionnaire had 11 sections, among which was the section on violence and unintentional injury. We selected questions for our analysis based on the literature we reviewed. The independent variables were derived from the following questions: How old are you? What is your sex? During the past 12 months, how often have you felt lonely? During the past 12 months, how often have you been so worried about something that you could not sleep at night? During the past 30 days, on how many days did you have at least one drink containing alcohol? During the past 30 days, on how many days did you miss classes or school without permission? During the past 12 months, did you ever seriously consider attempting suicide? The outcome variable was derived from the question: During the past 30 days, on how many days were you bullied? Study participants who reported having been bullied once or more were categorized as 1 for the outcome variable, otherwise, 0. Variables that had more than two possible responses were categorized in a similar way, except for the variable age that was categorized into: < 14, 14, 15, and 16+ years from its original categories of ≤11, 12, 13, 14, 15, 16+ years.

Completion of the questionnaire occurred within one class period varying between 30 and 40 minutes between schools. Twelve trained research assistants supervised the process.

**Data analysis**

Data analysis was performed using SPSS version 11.5 software. A weighting factor was used in the analysis to reflect the likelihood of sampling each student and to reduce bias by compensating for differing patterns of non response. We conducted bivariate logistic regression analyses in order to screen factors for further analysis. All significant factors which attained p<0.10 level in bivariate analyses were considered for further analysis. In order to estimate associations between independent factors and the outcome variable, a multivariate logistic regression analysis was conducted. We report prevalence of victimization from bullying as well as the unadjusted odds ratios (OR) and adjusted odds ratios (AOR) together with their 95% confidence intervals (CI).

## Results

**Psychosocial and behavioral description of the sample**

A total of 2136 students participated in the 2004 Zambia GSHS; and 1559 had information on whether they were bullied or not. Overall, 72.7% of the participants felt lonely; 70.1% were worried such that they could not sleep at night; and 41.7% consumed alcohol. Slightly over half (58.1%) of the participants missed classes without permission, and 31.8% of them seriously considered attempting suicide. Altogether, 62.8% (60.0% of male and 65.0% of female) participants reported having been bullied in the previous 30 days to the survey. Further description of the sample by gender is shown in Table 1.

**Table:** Table 1:** Psychosocial and behavioral description of the sample in the 2004 Zambia Global School-based Health Survey**

Factor	Total n* (%)**	Male n (%)	Female n (%)
Age (years)			
<14	419 (25.9)	156 (21.5)	263 (30.9)
14	375 (19.4)	156 (17.4)	219 (21.6)
15	498 (23.2)	260 (24.5)	238 (21.6)
16+	700 (31.6)	394 (36.5)	306 (25.9)
Loneliness			
Yes	1411 (72.7)	674 (70.5)	737 (75.3)
No	563 (27.3)	297 (29.5)	266 (24.7)
Worried			
Yes	1380 (70.1)	656 (69.0)	724 (71.3)
No	590 (29.9)	307 (31.0)	283 (28.7)
Consumed alcohol			
Yes	500 (41.7)	217 (38.5)	283 (45.1)
No	776 (58.3)	388 (61.5)	388 (54.9)
Missed classes			
Yes	956 (58.3)	463 (58.1)	493 (58.4)
No	746 (41.7)	365 (41.9)	381 (41.6)
Considered attempting suicide			
Yes	589 (31.8)	286 (31.6)	303 (32.0)
No	1294 (68.2)	633 (68.4)	661 (68.0)
Bullied			
Yes	588 (62.2)	314 (60.0)	274 (65.0)
No	897 (37.8)	431 (40.0)	466 (35.0)

* unweighted frequencies ** weighted percents

**Associations of psychosocial and behavioral factors with being bullied**

Table 2 shows associations of psychosocial and behavioral factors with victimization from bullying. All the factors considered in our analysis were significantly associated with being bullied in both bivariate and multivariate analyses. However, while the male sex was significantly positively associated with being bullied in multivariate analysis, it was significantly negatively associated with being bullied in a bivariate analysis.

**Table:** Table 2: **Associations of psychosocial and behavioral factors with victimization from bullying in bivariate and multivariate analyses**

Factor	Unadjusted Odds ratio (95% CI*)	Adjusted Odds ratio (95% CI)
Age (years)		
<14	1.09 (1.07, 1.10)	0.93 (0.91, 0.95)
14	1.12 (1.10, 1.13)	1.22 (1.19, 1.25)
15	1.06 (1.05, 1.08)	1.10 (1.08, 1.13)
16+	1	1
Gender		
Male	0.90 (0.89, 0.91)	1.07 (1.06, 1.09)
Female	1	1
Loneliness		
Yes	1.42 (1.41, 1.44)	1.24 (1.22, 1.26)
No	1	1
Worried		
Yes	1.33 (1.32, 1.34)	1.12 (1.11, 1.14)
No	1	1
Consumed alcohol		
Yes	3.23 (3.19, 3.28)	2.59 (2.55, 2.64)
No	1	1
Missed classes		
Yes	1.82 (1.80, 1.84)	1.30 (1.28, 1.32)
No	1	1
Considered attempting suicide		
Yes	1.32 (1.30, 1.33)	1.20 (1.18, 1.22)
No	1	1

CI* Confidence Interval

In multivariate analysis, we found that compared to participants of age 14 years or older, participants of age less than 14 years were less likely to be bullied. Male participants were 7% (AOR=1.07; 95%CI [1.06, 1.09]) more likely to be bullied compared to females. Participants who felt lonely or were worried were 24% (AOR=1.24; 95%CI [1.22, 1.26]) and 12% (AOR=1.12; 95%CI [1.11, 1.14]) more likely to have been bullied compared to participants who were not lonely or worried, respectively. Compared to participants who did not consume alcohol, participants who consumed alcohol were 2.59 (95%CI [2.55, 2.64]) times more likely to report having been bullied. Furthermore, participants who missed classes were 30% (AOR=1.30; 95%CI [1.28, 1.32]) more likely to report having been bullied compared to those who did not miss classes. Lastly, participants who considered committing suicide were 20% (AOR=1.20; 95%CI [1.18, 1.22]) more likely to report having been bullied compared to those who did not consider committing suicide.

## Discussion

In a cross-sectional national study of Zambian school-attending adolescents conducted in 2004, about two-thirds of the participants, i.e. 62.8% (60.0% of male and 65.0% of female) reported having been bullied in the previous 30 days to the survey. In a similar study in Venezuela, Muula et al^16^reported that 31.5% (37.0% of male and 27.0% of female) participants reported having been bullied in the previous 30 days. Out of a total of 2,348 in-school adolescents who participated in another similar survey in China, Hazemba et al.^17^ reported that 20% (23% of male, and 17% of female) respondents had been bullied in the previous 30 days to the survey. Thus, comparing our data to the Venezuelan and Chinese studies, we find that Zambian adolescents were more than twice as likely to report having been bullied. The variation in the victimization rate may be due to differences in perceptions of what bullying is across countries. Craig et al^12^suggest that the variation in the rates of victimization in different European countries partly reflects differences in the implementation of national policies and programs against bullying. In Zambia, there is no national policy against bullying. However, in some private schools, bullying is strictly prohibited warranting suspension or expulsion while in public schools that may not be the case. Furthermore, victimization may not be occurring in school yards but when students are on their way home. Such cases may occur when students are not picked up by their parents or guardians from school. Further studies are needed to establish where victimization takes place; and to evaluate the anti-bullying program in Zambia.

The finding that respondents of age less than 14 years were less likely to have been bullied compared to students aged 16 years or older suggests that younger children were less exposed to older children for possible victimization. Children of age less than 14 years are more likely to be in primary schools (Grades 1 to 7), and the older children in secondary schools (Grades 8 to 12). These schools tend to have different breaks or knocking off times suggesting fewer contacts between these age groups.

In this study, males were more likely to report being victimized than females, suggesting that male dominance over females may not be a major issue in Zambia among school-attending children. It may also just suggest males were more likely to be exposed to situations where bullying was likely compared to females. Although previous studies have reported similar findings,^1,5,17-19^in a recent cross-national study on bullying and victimization among adolescents in 40 countries, Craig et al found that the rates of bullying victimization were higher in girls in 29 out of 40 countries.^20^However, a study by Peskin et al^21^found no gender difference in victimization rates. We found that males were less likely to be bullied on bivariate analysis, and more likely to be bullied in a multivariate analysis. This may have been due to the interaction between gender and age. Older adolescents were more likely to be bullied. Of particular note was that there were older male respondents than females in the sample.

We found significant associations between loneliness and having worried thoughts on one hand and victimization from bullying on the other. Students who may be struggling emotionally (reported being lonely or worried) may also be more likely to fall victims to others who may identify them as vulnerable. On the other hand, students who may be victims may be more likely to feel lonely or worried as a consequence of their experience. A similar finding of a significant association between loneliness and victimization in 19 low- and middle income countries has been reported by Fleming and Jacobsen1 among 13-15 year-olds and Kim et al.^3^

The finding that alcohol consumption was related to victimization can be viewed as a coping strategy to ‘drink off’ the problem. Our finding of a significant association between alcohol use and victimization has also been reported by Fleming and Jacobsen^1^ among 13-15 year-olds. It is also possible that adolescents who drink alcohol are more exposed to situations or environment where bullying is more likely to occur than those who do not drink alcohol.

School children who may be bullied may miss classes in order to avoid being victimized. The finding in the current study that missing classes is associated with victimization from bullying has also been reported in Swaziland. ^11^Missing classes by victims would in turn lead them to poorly perform in school.

Students who seriously considered attempting suicide were also more likely to have reported having been victimized. While we do not know whether victimization was causative to these suicidal considerations, the finding that both these experiences are related is a matter of concern. A similar finding has been reported elsewhere.^1,9,10^Brunstein-Klomek et al^10^suggest that the association between victimization and suicidality is more than a correlate and studies should be conducted to identify causal paths between these factors.

**Limitations of the study**

Despite the fact that the current study was national, and therefore the findings could as well be a fair representation of the country among school-attending adolescents, the present study has a number of limitations. Firstly, data were self-reported; there is therefore the possibility of reporting misclassification bias. Secondly, students who were eligible to participate but were absent on the day the survey was conducted in their school, were not followed-up. If truant or students who miss school for any reason have different experiences compared to those who regularly attend school, our results could be biased. The survey also collected data only from school-based adolescents. Our findings may therefore not be representative of all adolescents in the country; especially among out of school adolescents. As with all cross-sectional studies, a relationship between independent and outcome variables is an association that may not be causal in nature. This is particularly so especially as the temporal sequence between the exposure (independent variable) and the outcome may not be known. Finally, while we controlled for the known confounder variables, there may have been some unmeasured variables that we may have missed. For example, Bowes et al ^22^have reported that school size, problems with neighbors, and family factors (e.g., child maltreatment, domestic violence) were associated with an increased risk for being a victim of bullying. Finally, from a sample of 2136 students, 1559 (73%) responded to the question on whether or not they were bullied. Comparing non- respondents to respondents with respect to the study factors, non-respondents tended to be younger than 14 years. No significant differences between the two groups were observed in the other study factors. Hence, the observed estimate for victimization from bullying may have been an over estimate of the true rate in Zambia, given that respondents below the age of 14 years were less likely to be bullied.

## Conclusion 

We have reported factors associated with victimization from bullying using the largest nationally representative sample ever conducted in Zambia among school-attending adolescents in grades 7 to 10. We found a prevalence of victimization which was much higher than reports from other parts of the world. Prevention and intervention strategies should take into consideration the factors that have been identified in this study.

**Authors’ Contributions**

SS conducted the data analysis and participated in the drafting of the manuscript. ASM drafted the first manuscript of the paper and contributed to the interpretation of the findings. ER participated in the interpretation of findings and manuscript preparation. All authors agreed to the final draft of the manuscript.
